# Prognostic value of CD155/TIGIT expression in patients with colorectal cancer

**DOI:** 10.1371/journal.pone.0265908

**Published:** 2022-03-24

**Authors:** Daisuke Murakami, Kenji Matsuda, Hiromitsu Iwamoto, Yasuyuki Mitani, Yuki Mizumoto, Yuki Nakamura, Ibu Matsuzaki, Ryuta Iwamoto, Yuichi Takahashi, Fumiyoshi Kojima, Shin-ichi Murata, Hiroki Yamaue

**Affiliations:** 1 Second Department of Surgery, Wakayama Medical University, Wakayama, Japan; 2 Department of Human Pathology, Wakayama Medical University, Wakayama, Japan; Osaka Medical Center for Cancer and Cardiovascular Diseases, JAPAN

## Abstract

**Introduction:**

The interaction of CD155 with its ligand, the T cell immunoreceptor with Ig and ITIM domains (TIGIT), is being studied owing to its potential to act as a target in the treatment of various solid tumors. However, the relationship between CD155 and TIGIT in colorectal cancer (CRC) prognosis is not known. We hypothesized that the TIGIT–CD155 pathway suppresses the attack of T cells on tumors, thereby affecting CRC prognosis.

**Methods:**

We examined the expression of CD155 and TIGIT using immunohistochemical staining in 100 consecutive patients with CRC who underwent complete resection of ≤Stage III tumors at Wakayama Medical University Hospital between January and December 2013. We assessed the correlation between CD155 and TIGIT expressions and prognosis as well as the clinicopathological background of CD155 and TIGIT.

**Results:**

Patients with high CD155 and TIGIT expressions showed worse prognosis than those with other levels of expression (p = 0.026). In multivariate analysis that also included the existing prognostic markers, high CD155 and TIGIT expressions were identified as independent poor prognostic factors.

**Conclusions:**

The interaction between CD155 and TIGIT possibly plays an important role in the immunological mechanism of CRC. Therefore, it may be possible to effectively predict the postoperative prognosis of CRC by evaluating the combined expression of CD155 and TIGIT. The study findings suggest that CD155 and TIGIT can predict clinical outcomes, thereby contributing to the personalized care of CRC.

## Introduction

Colorectal cancer (CRC), particularly colorectal adenocarcinoma, is an important neoplasm with high incidence and mortality [1]. The current treatment approach for CRC is tumorectomy at the early stage and chemotherapy and/or radiation therapy at the advanced stage [2]. In recent years, certain immune checkpoint molecules that belong to the CD28 superfamily, such as programmed cell death 1 (PD 1) and PD ligand 1 (PD-L1), have been reported as potential targets in CRC immunotherapy [3].

In the present study, we focused on the transmembrane protein CD155, which is a nectin-like molecule overexpressed in several cancers [4–6]. In malignant melanoma, CD155 deficiency upregulates the expression of DNAX accessory molecule 1 in CD8+ T cells and natural killer (NK) cells, which inhibit tumor growth and metastasis. Furthermore, in recent studies on hepatocellular carcinoma, CD155 was shown to be closely associated with disease exacerbation, indicating its potential for use as a prognostic marker [7, 8]. It has also been reported that CD155 functions as a prognostic factor of recurrence and overall survival in small cell lung cancer, pancreatic cancer, bile duct cancer, soft tissue sarcoma, and melanoma [9–13]. Moreover, several studies investigating tumor-expressing proteins have examined the role of the extrinsic regulators of cancer cells in the tumor microenvironment (TME) [14, 15]. For instance, the role of T cell immunoreceptor with Ig and ITIM domains (TIGIT) in T cell depletion during binding with CD155 is a focus of immune checkpoint research [16]. In particular, TIGIT overexpression is observed in several malignant tumors, including gastric cancer, noninvasive ductal carcinoma, and melanoma [17–19]. However, limited studies have been conducted on the interaction between CD155 and TIGIT in the context of CRC.

We hypothesized that the TIGIT–CD155 pathway suppresses T cell attack on tumors, thereby affecting prognosis. Thus, this study aimed to clarify the clinical importance of CD155 and TIGIT in CRC.

## Materials and methods

### Patients

The subjects comprised 100 consecutive patients who underwent surgical resection for ≤Stage III CRC at Wakayama Medical University Hospital between January and December 2013. We examined the patients’ clinicopathological features, including age, sex, histological grade, TNM staging, follow-up data, neutrophil/lymphocyte ratio (NLR), lymphocyte/monocyte ratio (LMR), modified Glasgow prognostic score (mGPS), CA19-9, and carcinoembryonic antigen (CEA). We did not use surgical specimens and clinicopathological data for research purposes. The study was approved by the Research Ethics Committee of Wakayama Medical University Hospital (approval number: 2747), and patients’ verbal consent was obtained. We recorded the patients’ clinicopathological features in the patient chart. Clinical specimens were stored and processed in the Department of Human Pathology, whereas clinical pathological data were stored in the Second Department of Surgery, Wakayama Medical University. All cases were followed up for >5 years and a maximum of 7 years.

### Immunohistochemical staining

For the immunohistochemical staining of CD155 and TIGIT, 4-μm tissue sections, which were obtained by treating surgically removed patient tissues with 10% buffered formalin and paraffin, were used. The following primary antibodies were used: mouse monoclonal antibody CD155 (1:50, catalog no. sc-514623; Santa Cruz Biotechnology, CA, USA) and TIGIT (1:25, catalog no. DIA-TG1; ONCODIANOVA, Hamburg, Germany); the antibodies were diluted with a primary antibody diluent (Leica Microsystems, Wetzlar, Germany).

**Fig 1:**
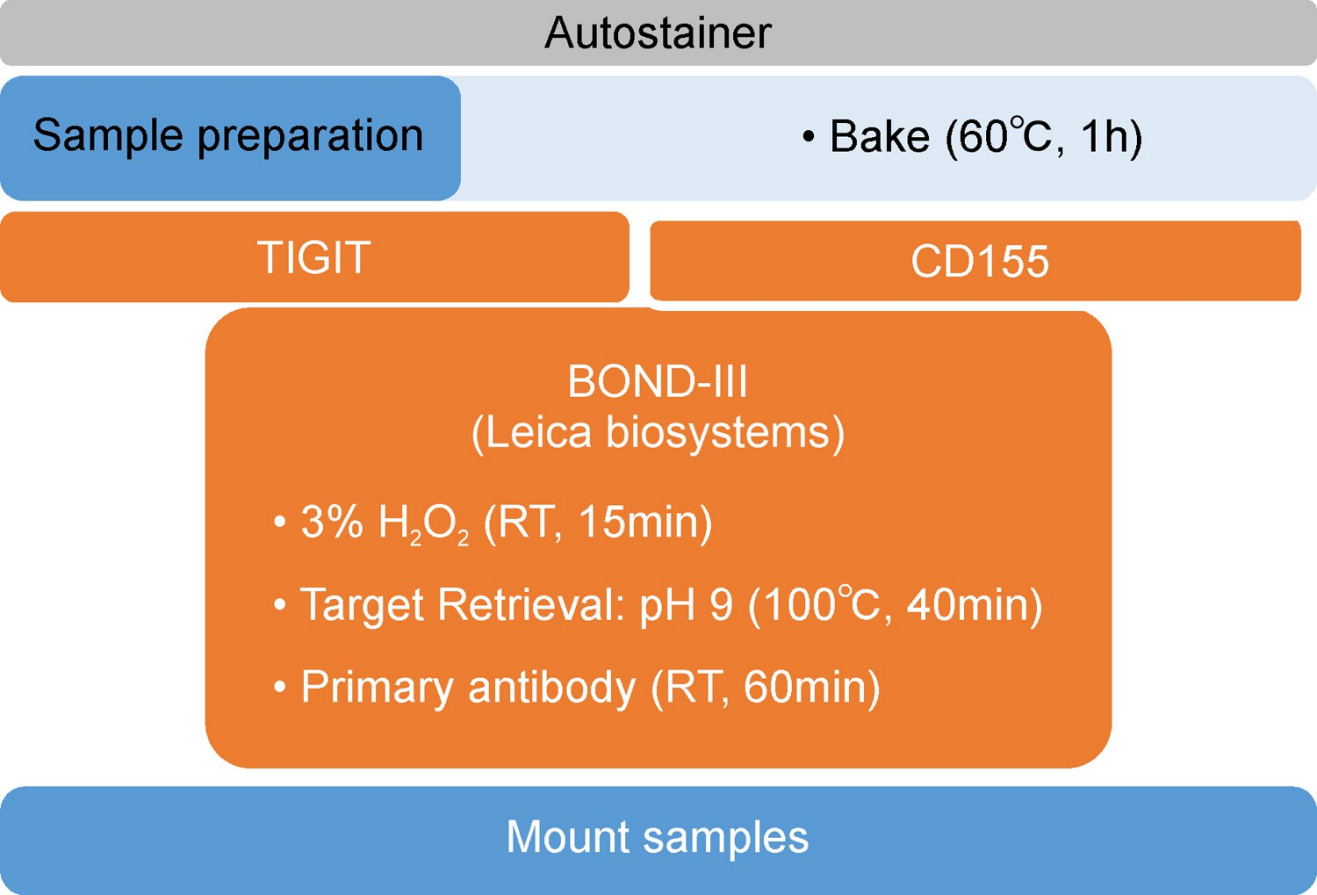
An overview of immunohistochemical staining of CD155 and Ig and ITIM domains (TIGIT).

BOND-III Automated Immunostainer (Leica Microsystems) was used for staining. For the antigen activation method, CD155 was thermally treated (at 100°C) for 15 min with BOND Epitope Retrieval Solution 1 (Leica Microsystems), whereas TIGIT was thermally treated for 30 min with BOND Epitope Retrieval Solution 2 (Leica Microsystems). The treatment time for the primary antibody was 30 min. The BOND Polymer Refine Detection Kit (Leica Microsystems) was used for DAB staining. Fig 1 shows the outline of the immunohistochemical staining procedure.

### Immunohistochemical evaluation

Immunohistochemical assessment was performed by two independent pathologists using an optical microscope (Olympus). The CD155 immunostaining intensity of the tumor was determined at a high-power field (200×). In particular, positive cells were defined as those in which the cytoplasm was intensely and clearly stained brown. Samples comprising ≥50% CD155+ tumor cells were classified as CD155-high, whereas those comprising <50% CD155+ tumor cells were classified as CD155-low [10]. Lymphocytes within the tumor were graded as follows: 0 = ≤10% positive lymphocytes; 1 = 11%–20% positive lymphocytes; 2 = 21%–50% positive lymphocytes; 3 = ≥50% positive lymphocytes; we classified 0 as TIGIT-low and 1–3 as TIGIT-high [20].

### Statistical analyses

All statistical analyses were performed using BellCurve for Excel (Social Survey Research Information Co., Ltd). The relationship between CD155 expression in tumor cells, TIGIT expression in tumor-infiltrating immune cells, and clinicopathological features were analyzed via chi-square test. The survival rate was analyzed using the Kaplan–Meier method, and the significance of the differences in the survival rate was evaluated using the log-rank test. The variation effect in terms of overall survival was calculated using the Cox regression model. P-values < 0.05 were considered statistically significant.

## Results

A total of 100 patients with CRC who underwent radical resection were included in this study. Tables 1–3 summarize patient background. A total of 53 (53%) patients were aged > 70 years, and 47 (47%) patients were aged < 70 years. The study group comprised 55 males (55%) and 45 females (45%). With regard to T stage, 15 patients belonged to Tis/1a/1b and 85 belonged to T2/3/4. With regard to N stage, 61 patients belonged to N0 and 39 belonged to N1/2/3. The tumor site was the right colon in 26 cases and the left colon in 74 cases. The histological grade was well-differentiated adenocarcinoma (tub1) in 40 patients and other histological grades in the remaining 60 patients. The optimum cutoff values for NLR and LMR were 2.85 and 2.82, respectively. A total of 62 patients had an NLR of ≤2.85 and 38 had an NLR of >2.85. Eleven patients had an LMR of ≤2.82 and 89 had an LMR of >2.82. A total of 79 patients had an mGPS of 0 and 21 had an mGPS of 1 or 2. Further, 63 patients had a preoperative CEA value of ≤5 ng/mL and 37 had a preoperative CEA value of >5 ng/mL. Finally, 93 patients had a preoperative CA19-9 value of ≤37 U/mL and 7 had a preoperative CA19-9 value of >37 U/mL.

**Table 1:** Patient background and CD155 expression level.

Category	CD155-high	CD155-low	P-value
**Age (≥70/<70)**	43/25	10/22	0.589
**Sex**			0.490
** Male**	39	16	
** Female**	29	16	
**T stage**			0.904
** Tis/T1a/T1b**	10	5	
** T2/T3/T4a/T4b**	58	27	
**N stage**			0.126
** N0**	38	23	
** N1/N2/N3**	30	9	
**Location**			0.875
** Right**	18	8	
** Left**	50	24	
**Histological grade**			0.431
** Tub1**	29	11	
** Others**	39	21	
**NLR**			0.304
** ≤2.85**	40	22	
** >2.85**	28	10	
**LMR**			0.722
** ≤2.82**	8	26	
** >2.82**	60	6	
**mGPS**			0.705
** 0**	53	26	
** 1 or 2**	15	6	
**CEA**			0.607
** ≤5 ng/mL**	44	19	
** >5 ng/mL**	24	13	
**CA19-9**			0.523
** ≤37 U/mL**	64	29	
** >37 U/mL**	4	3	

**Table 2:** Patient background and TGIT expression level.

Category	TIGIT-high	TIGIT-low	P-value
**Age (≥70/<70)**	46/33	7/14	0.042
**Sex**			0.824
** Male**	43	12	
** Female**	36	9	
**T stage**			0.954
** Tis/T1a/T1b**	12	5	
** T2/T3/T4a/T4b**	67	27	
**N stage**			0.179
** N0**	46	23	
** N1/N2/N3**	33	9	
**Location**			0.155
** Right**	18	8	
** Left**	61	13	
**Histological grade**			0.841
** Tub1**	32	8	
** Others**	47	13	
**NLR**			0.606
** ≤2.85**	50	12	
** >2.85**	29	9	
**LMR**			0.588
** ≤2.82**	8	3	
** >2.82**	71	18	
**mGPS**			0.338
** 0**	64	15	
** 1 or 2**	15	6	
**CEA**			0.532
** ≤5 ng/mL**	51	12	
** >5 ng/mL**	28	9	
**CA19-9**			0.141
** ≤37 U/mL**	75	18	
** >37 U/mL**	4	3	

**Table 3:** Patient background and CD155/TGIT expression.

Category	CD155/TIGIT-high	Others	P-value
**Age (≥70/<70)**	40/24	23/13	0.890
**Sex**			0.738
** Male**	36	19	
** Female**	28	17	
**T stage**			0.816
** Tis/T1a/T1b**	10	5	
** T2/T3/T4a/T4b**	54	31	
**N stage**			0.194
** N0**	36	25	
** N1/N2/N3**	28	11	
**Location**			0.761
** Right**	16	10	
** Left**	48	26	
**Histological grade**			0.307
** Tub1**	28	12	
** Others**	36	24	
**NLR**			0.471
** ≤2.85**	38	24	
** >2.85**	26	12	
**LMR**			0.979
** ≤2.82**	7	4	
** >2.82**	57	32	
**mGPS**			0.822
** 0**	51	28	
** 1or2**	13	8	
**CEA**			0.248
** ≤5 ng/mL**	43	20	
** >5 ng/mL**	21	16	
**CA19-9**			0.227
** ≤37 U/mL**	61	32	
** >37 U/mL**	3	4	

**Fig 2:**
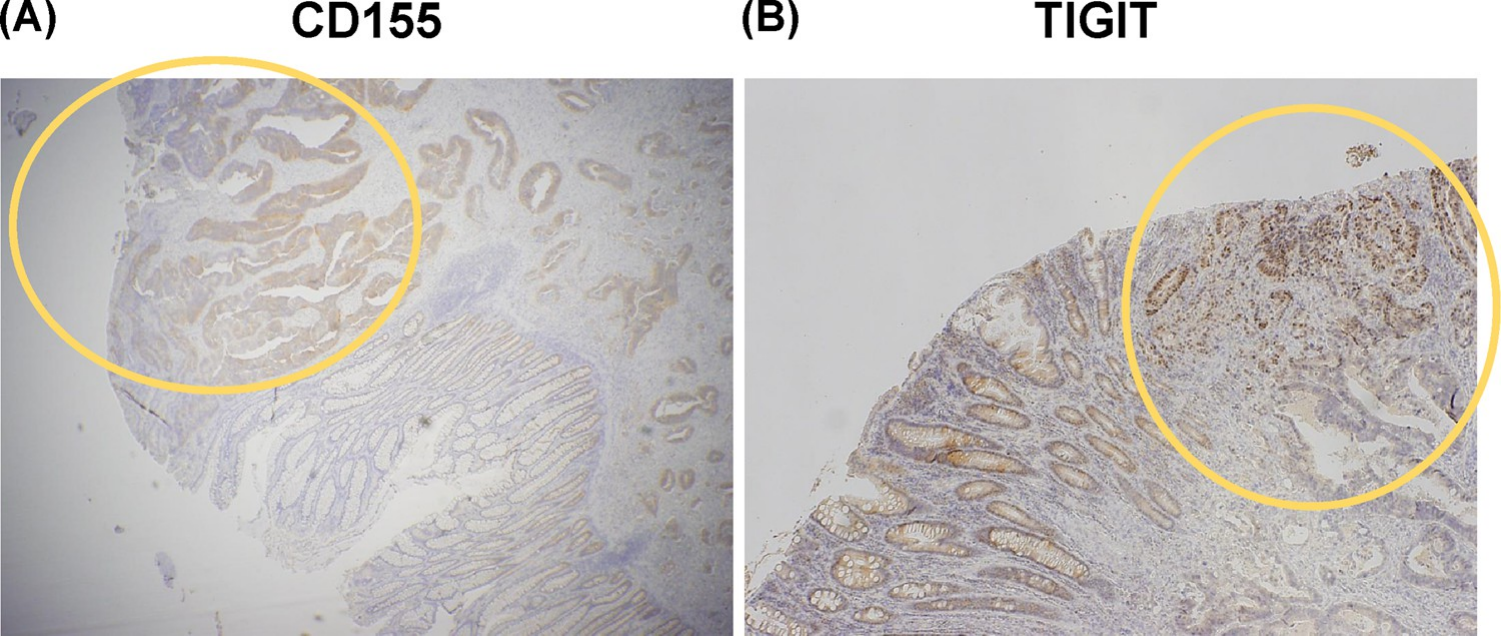
(A) CD155 expression in colorectal cancer and adjacent tissues. The yellow circle is the tumor area. A normal ductal area was observed. CD155 was expressed in malignant epithelial cells of colorectal cancer. **(B)** TIGIT expression in lymphocytes in colorectal cancer and adjacent tissues. The yellow circle is the tumor area. A normal ductal area was observed. TIGIT was expressed in lymphocytes in colorectal cancer tissues.

CD155 was expressed in malignant CRC epithelial cells but not in normal glandular epithelium (Fig 2A). We also found that TIGIT was highly expressed in the lymphocytes of CRC tissues than in the lymphocytes of adjacent tissues (Fig 2B).

**Fig 3:**
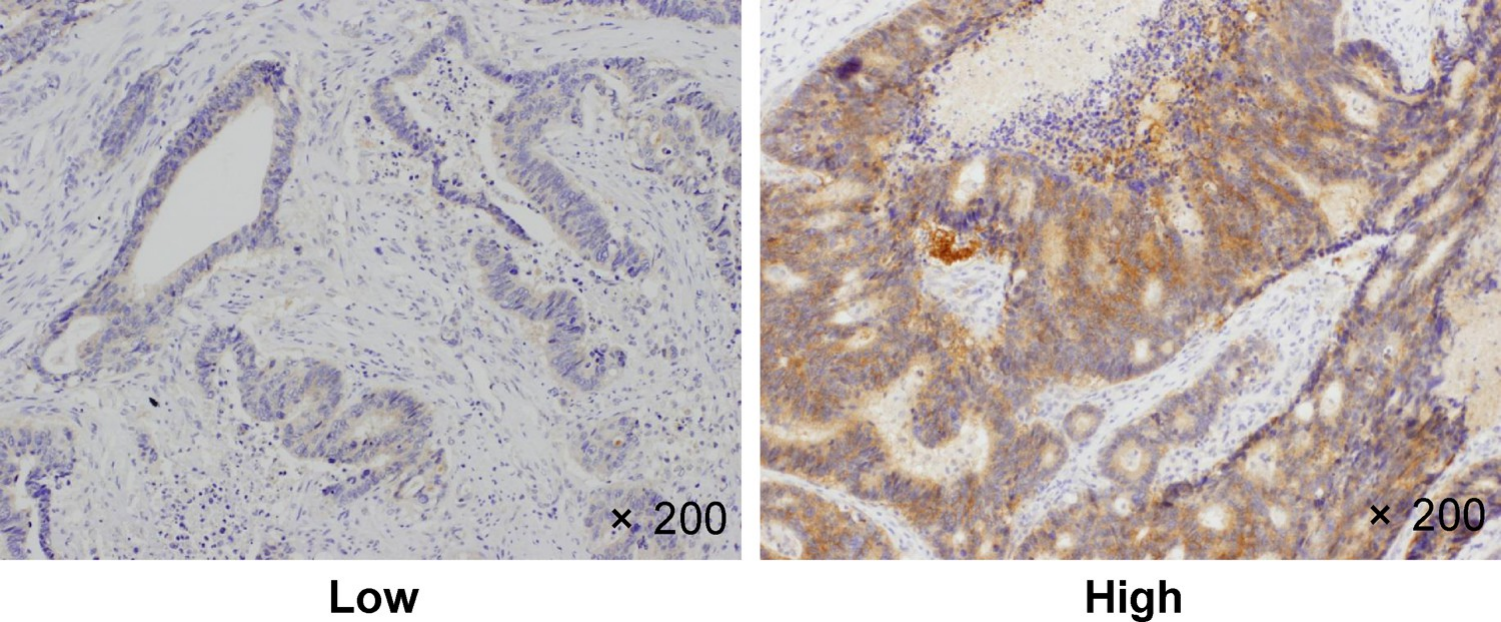
CD155 immunostaining. CD155 expression in human colorectal cancer tissues. Representative image showing high and low CD155 expressions. Original magnification, ×200.

**Fig 4:**
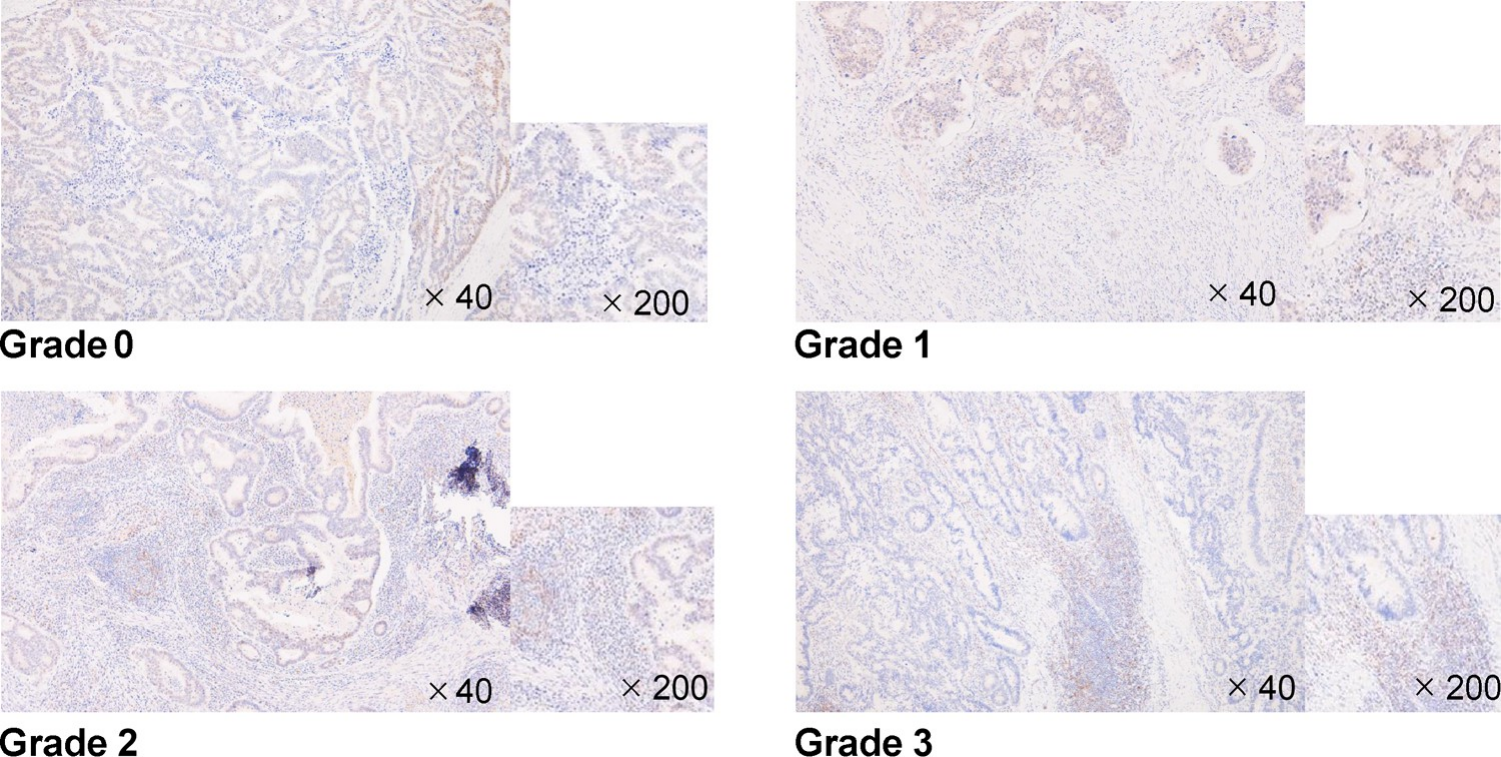
TIGIT immunostaining. TIGIT expression in colorectal cancer (CRC). TIGIT expression in human CRC tissues. Representative case with high (grade 1–3) and low (grade 0) TIGIT expression.

Figs 3 and 4 show the representative microscopic images of tumor-infiltrating lymphocytes (TILs) in CRC tissues stained with anti-CD155 and anti-TIGIT antibodies, respectively. Tables 1–3 show the overall distribution of CD155- and TIGIT-positive cells. In terms of CD155 expression, 68 patients (68%) had high expression and 32 (32%) had low expression. With regard to TIGIT expression, 79 patients (79%) had high expression and 21 (21%) had low expression. With regard to the combined expression of CD155 and TIGIT, 64 patients (64%) had high expression and 36 (36%) had other expression. All covariates (age, gender, T stage, N stage, location, differentiation, NLR, LMR, mGPS, CEA value, and CA19-9 value) were not associated with the expression of CD155 and TIGIT.

**Fig 5:**
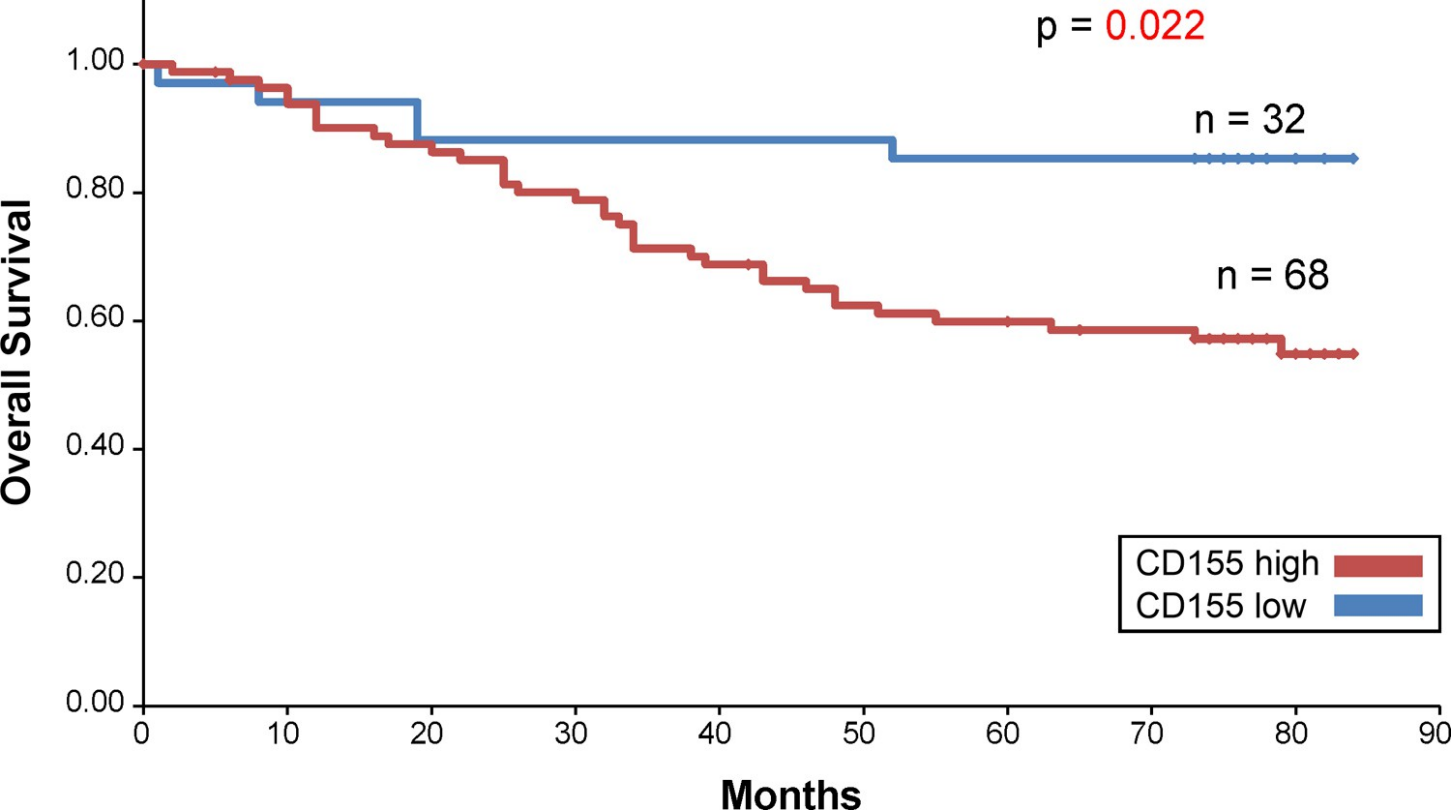
Overall survival curve for CD155.

**Fig 6:**
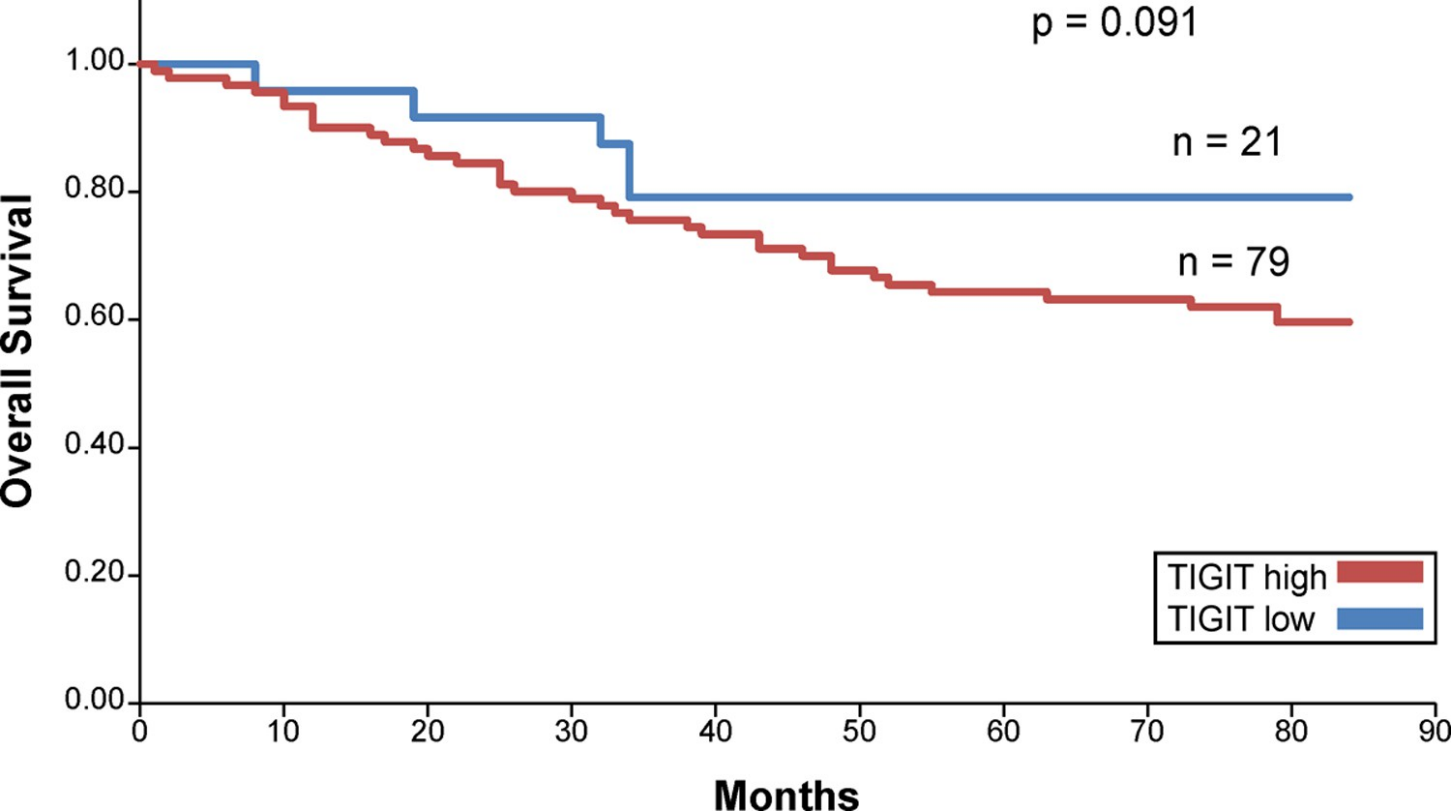
Overall survival curve for TIGIT.

**Fig 7:**
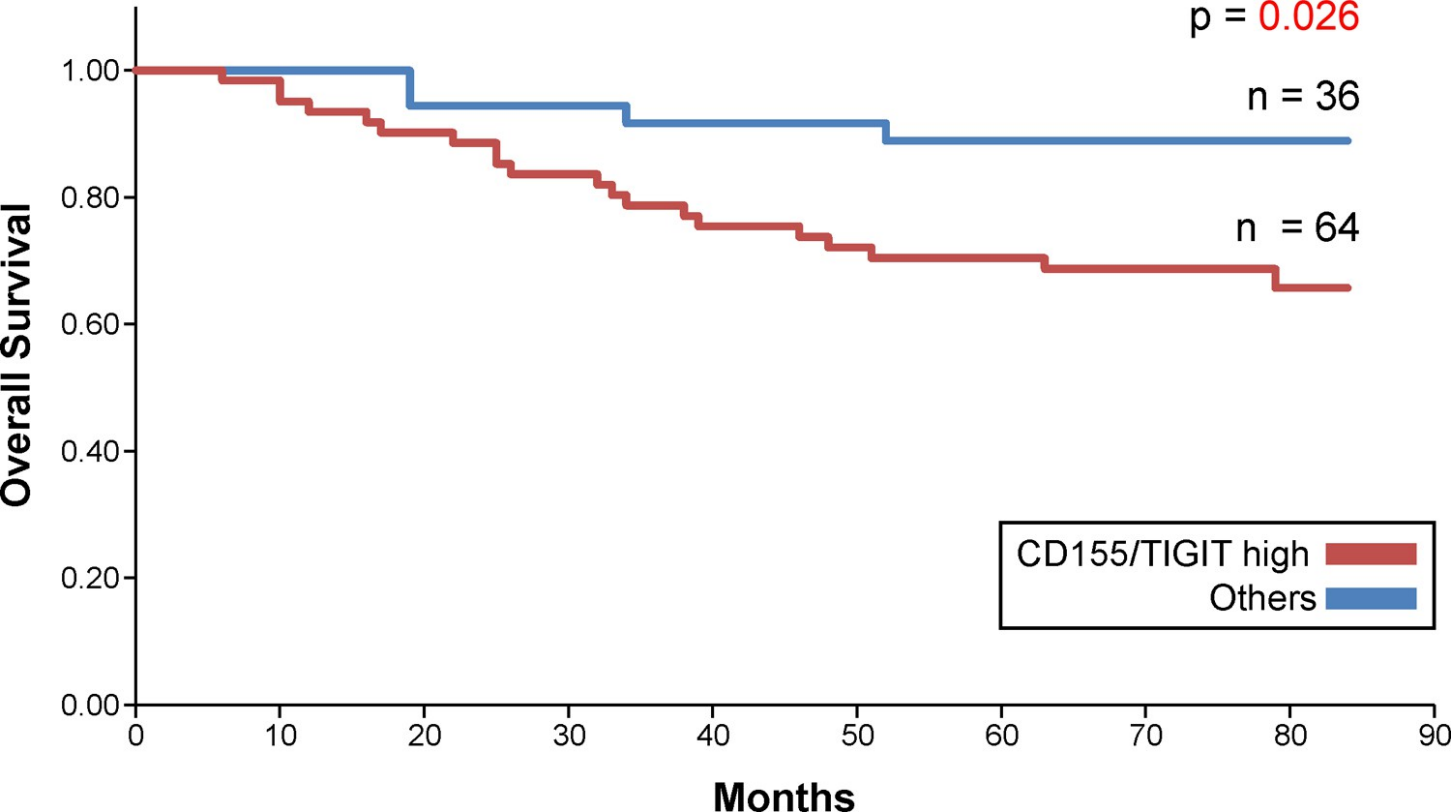
Overall survival curve for CD155 and TIGIT.

The Kaplan–Meier survival curve revealed that patients with high CD155 expression had short overall survival (OS) (Fig 5). No significant difference was observed in the survival curves of the high and low TIGIT expression groups (Fig 6). In addition, when the prognostic value of the combined expression of CD155 and TIGIT in determining OS was analyzed, the OS of patients with high CD155 and TIGIT expression was found to be the lowest (Fig 7). The 5-year survival rate for patients with high CD155 expression was 70.7%, and the 5-year survival rate for those with low CD155 expression was 90.6%. The 5-year survival rate for patients with high TIGIT expression was 73.6%, and the 5-year survival rate for those with low CD155 expression was 90.5%. In addition, the 5-year survival rate for patients with high expression of both CD155 and TIGIT was 70.4%, and the 5-year survival rate for other patients was 88.9%.

Univariate Cox proportional hazards regression analysis for OS (Table 4) showed that patients with high CD155 and TIGIT expressions had a higher risk of death than other patients [hazard ratio (HR), 3.272; 95% confidence interval, 1.117–9.589; p = 0.031]. As per multivariate analysis (Table 5), in addition to lymph node metastasis and elevated CEA, high expressions of CD155 and TIGIT were independent prognostic predictors in patients with CRC. Taken together, these results suggest that a combined analysis of CD155 and TIGIT expressions represents an effective prognostic predictor of CRC.

**Table 4:** Univariate analysis for overall survival.

Category	HR (95% CI)	P-value
**Age (≥70)**	2.497 (1.035–6.023)	0.042
**Sex (male)**	1.722 (0.737–4.026)	0.210
**T stage (T2, T3, T4a, T4b)**	2.084 (0490–8.867)	0.320
**N stage (N1, N2, N3)**	3.187 (1.393–7.291)	0.006
**Location (right)**	2.681 (0.800–8.992)	0.110
**Histological grade (tub1)**	0.689 (0.324–1.466)	0.333
**CD155/TIGIT (high/high)**	3.272 (1.117–9.589)	0.031
**CEA (>5 ng/mL)**	2.490 (1.114–5.566)	0.026
**CA19-9 (≥37 U/mL)**	1.111 (0.261–4.731)	0.887
**NLR (≥2.85)**	2.709 (1.202–6.101)	0.016
**LMR (≤2.82)**	0.252 (0.100–0.639)	0.004
**Modified GPS (1/2)**	3.225 (1.431–7.264)	0.005

**Table 5:** Multivariate analysis for overall survival.

Category	HR (95% CI)	P-value
**Age (≥70)**	2.299 (0.934–5.786)	0.077
**N stage (N1, N2, N3)**	3.623 (1.449–9.061)	0.006
**CD155/TIGIT (high/high)**	3.944 (1.221–12.736)	0.022
**CEA (>5 ng/mL)**	2.716 (1.148–6.429)	0.023
**NLR (≥2.85)**	1.201 (0.460–3.132)	0.708
**LMR (≤2.82)**	0.354 (0.112–1.117)	0.077
**Modified GPS (1/2)**	2.030 (0.774–5.324)	0.150

## Discussion

Tumor cells are involved with inhibitory T cell receptors; by weakening T cell function in TME and expressing several ligands, tumors escape from immune surveillance. Therefore, increasing the amount of TILs is important for improving anticancer treatment. Therefore, the use of immune checkpoint inhibitors, including cytotoxic T lymphocyte-associated antigen 4 and PD-1, has been evaluated in clinical trials and shown to prolong patient survival [21–24]. However, so far, their clinical tumor suppression effects appear to be limited. Therefore, we believe that it is necessary to search for new therapeutic targets. In addition, the role of biochemical and molecular markers in the diagnosis, prognosis, and treatment of malignant tumors in patients with various carcinomas is currently being emphasized [25, 26]. Hence, we focused on the recently reported CD155–TIGIT pathway. It has been reported that the combined expression level of CD155 and TIGIT correlates with the prognosis of lung adenocarcinoma, gastric cancer, hepatocellular carcinoma, and head and neck squamous cell carcinoma [27–30].

Few studies have examined the CRC-related prognosis using immunohistochemical staining, and it was hypothesized that similar correlation exists for CRC. In the present study, we evaluated the clinical importance of a recently discovered immunosuppressive ligand, CD155, as well as TIGIT for treating CRC.

Studies have shown that CD155 plays an extremely important role in the development of various malignant tumors, including melanoma, pancreatic cancer, human bile duct cancer, glioblastoma, breast cancer, and primary small cell carcinoma of the esophagus [7, 11, 31–33]. In the present study, CD155 overexpression was observed in 68% of tissues samples collected from patients with CRC, and it was clearly higher in the tumor regions than in the normal regions. Furthermore, CD155-high patients had notably worse prognosis than CD155-low patients. Studies have shown that the expression of CD155 contributes to the postoperative prognosis of patients with malignant tumors, including lung adenocarcinoma and soft tissue sarcoma [12, 29, 34]. Our data further highlight the importance of CD155 as a therapeutic target in CRC. However, the immunological role of CD155 is complex, and many immune cells other than CRC cells could be affected by treatments that target CD155. Thus, given that there is a chance that acquired immune deficiency, autoimmune disorders, or unknown adverse events could be induced by CD155-targeted therapy, further study is necessary.

TIGIT prevents autoimmune reaction and functions as a checkpoint molecule that helps balance the immune system [35]. In this study, TGIT expression was clearly higher in the tumor regions than in the normal regions. Although the expression of TIGIT alone was not associated with prognosis, prognosis was poor in patients with high expression levels of both CD155 and TIGIT.

In multivariate analysis, high expression of CD155 and TIGIT was also proved to be a prognostic marker along with other known prognostic markers. This finding suggests that poor postoperative prognosis can be determined by analyzing the combined expression of CD155 and TIGIT.

To date, limited studies have evaluated the expression of CD155 and TIGIT using immunohistochemical staining in patients with CRC. In the present study, we confirmed a significant difference in the survival curves based on the intensity of CD155 expression but not in those based on the expression of TIGIT alone. These differences are likely caused by other factors in TME, which could not be elucidated by immunohistochemical staining alone. In addition, Qu et al. showed that the high expression of CD155 is associated with good prognosis, which is contrary to our findings [36]. This suggests that it is necessary to standardize CD155 and TIGIT scoring patterns and detection methods to achieve a more reliable and reproducible prediction using the prognostic markers of CRC and other cancers. Furthermore in the multivariate analysis, significant differences were found in the N stages and CEA levels in addition to the high expression of both CD155 and TIGIT. For patients in the advanced stages of CRC, more research is needed to eliminate the effects of bias due to patient treatments.

In conclusion, by analyzing the combined expression of CD155 and TIGIT, it may be possible to effectively predict the postoperative prognosis of patients with CRC. Thus, this combined expression may have potential as a target for CRC therapy. Anti-TIGIT drugs are currently being assessed in clinical trials. Furthermore, the group with high CD155 and TIGIT expressions showed poor prognosis. Thus, CD155 and TIGIT expressions may be useful prognostic markers. The study findings suggest that CD155 and TIGIT can predict clinical outcomes, thereby contributing to the development of personalized care for patients with CRC.
